# Biological background and biomechanical support explain similar effect in treatment of scoliosis and kyphosis with TLI bracing

**DOI:** 10.1186/1748-7161-8-S1-P4

**Published:** 2013-06-03

**Authors:** PJM Van Loon

**Affiliations:** 1 Orthopedium Delft, Delft, The Netherlands

## Background

Practice in bracing for scoliosis did not lead to consensus in methods. Sound etiologic factors like insight in growth will give better conservative therapy. In pre-Pubmed literature, concepts were found to formulate and apply consequences in treatment strategy.

## Characteristics of progress in earlier research periods

Pre WW I literature showed doubtless relation between musculoskeletal function and morphogenesis, and knowledge on good and bad posture. Sitting of children got evidence a etiologic factor. After WW II all was based on the AP X-ray, and clinical aspects were forgotten. Bracing techniques start to prevent lordosis in scoliosis.

## Opening the black box on morphogenesis: the work of Milan Roth

The work of Milan Roth was rediscovered 1. In his concepts of the “Nervous Skeleton” an important science on neurovertebral and neuro-osseous growth relations and the tension induced possibility of incongruence of growth show that a “short cord” can indeed cause scoliosis. Recent studies with MRI confirm much of this. Growth (muscular force) has to create wedged vertebral bodies in hindered stretch growth of the cord. Increased tension in the cord will give the easily assessable tension in muscles. MRI will fill the gap in visualization of the cord, the vertebrae, discs, joints and the muscles involved in one time.

## Can the proven instant correction be transformed in structural corrective growth?

On two usable issues for durable correction: the existence of a thoracolumbar kyphosis in scoliosis, confirmed by Ni et al. 15, and the possibility to correct double curved scoliosis by applying a symmetrical lordotic (and thus extending) force at the TL joint, the concept of Thoracolumbar Lordotic Intervention (TLI) was developed. By forcing the erecting muscles in their normal track the feared instability in thoracic lordosis is overcome by proper tension on muscles to overcome flabbiness. We have to disagree with Dickson and followers on their fear for lordosis. The remaining potential of (fast) spinal growth in the growth (spurt) could then act as a helpful ally in this.

## The design of a corrective brace

The TLI brace applies a complete controlled lordosis, and strain on the erector trunci is given, so normal growth forces are being brought back in their anatomical tracts. It also prevents flexion, the rapidly increased and most prevailing “posture” in modern life of children. We brought promising results in a pilot study. Because of bringing a good posture, a good compliance can be expected.
